# Cytokine storm and histopathological findings in 60 cases of COVID-19-related death: from viral load research to immunohistochemical quantification of major players IL-1β, IL-6, IL-15 and TNF-α

**DOI:** 10.1007/s12024-021-00414-9

**Published:** 2021-08-31

**Authors:** Paolo Frisoni, Margherita Neri, Stefano D’Errico, Letizia Alfieri, Diana Bonuccelli, Mariano Cingolani, Marco Di Paolo, Rosa Maria Gaudio, Maurizio Lestani, Matteo Marti, Massimo Martelloni, Carlo Moreschi, Alessandro Santurro, Matteo Scopetti, Ombretta Turriziani, Martina Zanon, Roberto Scendoni, Paola Frati, Vittorio Fineschi

**Affiliations:** 1 Department of Medical Sciences, University of Ferrara, Ferrara, Italy; 2 Department of Surgical, Medical and Health Sciences, University of Trieste, Trieste, Italy; 3 Department of Legal Medicine, Territorial Unit USL Toscana Nordovest Lucca, Pisa, Italy; 4 Department of Law, Institute of Legal Medicine, University of Macerata, Macerata, Italy; 5 Department of Surgical Pathology, Medical, Molecular and Critical Area, Institute of Legal Medicine, University of Pisa, 56126 Pisa, PI Italy; 6 Department of Translational Medicine, University of Ferrara, Ferrara, Italy; 7 Pathology Unit, Territorial Unit ULSS 7 Pedemontana, Alto Vicentino Hospital, Thiene, Italy; 8 Department of Medical Area (DAME), University of Udine, Udine, Italy; 9 Department of Anatomical, Histological, Forensic and Orthopaedic Sciences (SAIMLAL), Sapienza University of Rome, Rome, Italy; 10 Department of Molecular Medicine, Laboratory of Virology, Sapienza University of Rome, Rome, Italy

**Keywords:** COVID-19 related-death, Autopsy, SARS-CoV-2, RT-PCR, Pneumonia, Cytokines, Immunohistochemistry

## Abstract

This study involves the histological analysis of samples taken during autopsies in cases of COVID-19 related death to evaluate the inflammatory cytokine response and the tissue localization of the virus in various organs. In all the selected cases, SARS-CoV-2 RT-PCR on swabs collected from the upper (nasopharynx and oropharynx) and/or the lower respiratory (trachea and primary bronchi) tracts were positive. Tissue localization of SARS-CoV-2 was detected using antibodies against the nucleoprotein and the spike protein. Overall, we tested the hypothesis that the overexpression of proinflammatory cytokines plays an important role in the development of COVID-19-associated pneumonia by estimating the expression of multiple cytokines (IL-1β, IL-6, IL-10, IL-15, TNF-α, and MCP-1), inflammatory cells (CD4, CD8, CD20, and CD45), and fibrinogen. Immunohistochemical staining showed that endothelial cells expressed IL-1β in lung samples obtained from the COVID-19 group (p < 0.001). Similarly, alveolar capillary endothelial cells showed strong and diffuse immunoreactivity for IL-6 and IL-15 in the COVID-19 group (p < 0.001). TNF-α showed a higher immunoreactivity in the COVID-19 group than in the control group (p < 0.001). CD8 + T cells where more numerous in the lung samples obtained from the COVID-19 group (p < 0.001). Current evidence suggests that a cytokine storm is the major cause of acute respiratory distress syndrome (ARDS) and multiple organ failure and is consistently linked with fatal outcomes.

## Introduction

On December 2019 the China Health Authority alerted the World Health Organization (WHO) about several cases of pneumonia with unknown etiology [1–5]. Laboratory diagnosis of a new disease, termed coronavirus disease 2019 (COVID-19), was performed using throat swab samples of 41 patients hospitalized on January 2, 2020 [6–12].

On March 11, 2020, the WHO characterized the COVID-19 outbreak as a pandemic on the basis of its alarming spread and severity [13–16]. The WHO classified the causal agent of COVID-19, called the severe acute respiratory syndrome coronavirus 2 (SARS-CoV-2). Taxonomically, SARS-CoV-2 has been classified as a member of the species SARS-related coronavirus (SARSr-CoV) in the genus betacoronavirus (βCoV) of the family Coronaviridae [17]. A closely related SARSr-CoV genome sequence, RaTG13, which shares a 96% whole-genome sequence identity with SARS-CoV-2, has been identified [18]. The SARS-CoV-2 genetic sequence showed about 79% and 50% similarity with severe acute respiratory syndrome coronavirus and Middle East respiratory syndrome–related coronavirus, respectively [19]. The occurrence of infections between families supported the idea that droplets, contact, and aerosols were the probable routes of person-to-person transmission; transmission via the gastrointestinal system was also proposed as a possible route [20, 21]. Human lung epithelial cells have been indicated as a major target of the coronavirus. The receptor-binding domain of the viral spike protein interacts with the receptor of cellular angiotensin-converting enzyme 2 (ACE-2) [22–24]. In the early stages of the infection, patients are asymptomatic or mildly symptomatic, wherein they exhibit symptoms of fever, cough, fatigue, headache, hemoptysis, and diarrhea triggered by the initial local inflammatory response. In this phase, the virus infiltrates and damages the lung parenchyma progressively, and when the host inflammatory response continues to amplify, systemic inflammation damages other organs, leading to conditions such as acute kidney injury [25]. A cascade of biomolecular events occurs in an intricate network after exposure infection of SARS-COV-2 including the production of interleukins 1β, 6, 10 (IL-1β, IL-6, IL-10, MCP-1), and tumor necrosis factor-α (TNF-α). These molecules have a various set of functions. A proinflammatory behavior is reported for TNF-α, IL-1β and IL-6, which are important mediators of acute inflammatory response, such as for the recruitment of neutrophil leukocytes. Other molecules recognized with an immunosuppressant role include IL-10, which inhibits cytokine production and receptor expression.

Autopsy has been used as the gold standard for identifying the cause of death in COVID-19 cases [26–31], and several techniques have been recommended for the safety of pathologists and to reduce the risk of infection during autopsy [32–39]. Despite these recommendations, autopsies in COVID-19 cases are often limited to biopsies or minimally invasive thoracotomies [40–46]. Craniotomies, and dissection of the central nervous system is generally avoided to minimize the risk of exposure to aerosols [47–49]. Finally, only a few cases of complete postmortem investigations in these cases have been reported [50–80].

The aim of this study was to clarify the correlation between infection due to SARS-COV-2 and the inflammatory response, and to investigate the expression of cytokines such as TNF-α, IL-1β, IL-6, MCP-1, IL-10, IL-15, and leukocyte marker (CD 4, CD 8, CD20, CD 45), in an attempt to verify and define the role and expression of cytokines and mechanisms of cell death triggered in cases of COVID-19 deaths. We performed both immunohistochemical analysis and electron microscopy to analytically evaluate the infection status and its impact on various organs.

## Materials and methods

This study was approved (N 342/2020/Oss/AOUFe0) on April 7th, 2020 by the competent Ethic Committee (CE-AVEC: Comitato Etico di Area Vasta Emilia Centro della Regione Emilia-Romagna) according to the Helsinki Declaration of 1975 and according to the Italian law.

### Case selection

A total of 60 COVID-19-positive subjects were included (Group 1); the demographic data are shown in Table 1. As controls (Group 2), we selected a total of 20 subjects, who died of multi organ failure from polytrauma (n = 10) and gunshot head injuries (n = 10) prior to 2018, with an average survival of about 10 days in intensive care wards. The case exclusion criterion was the presence of concomitant known infectious lung diseases. Samples were anonymized by assigning them an alphanumeric code

**Table 1:** Demographics and clinical characteristics of the COVID 19 positive cases (Group 1) and control cases (Group 2)

**Demographics and clinical characteristics of the cases (N = 60)**						
Gender		*M* 28			*F* 32	
Age	Total	*20–40 years*	*40–50 years*	*50–60 years*	*60–70 years*	> *70 years*
		1	2	5	8	44
No comorbidity known	6	1	1	1	1	2
Arterial Hypertension	33		1	1	2	29
Malignancy	8		1	1	2	4
Chronic Kidney disease	10					10
Overweight	3		1		1	1
Diabetes Mellitus	11		1		3	7
Chronic pulmonary disease	20		1		1	18
**Demographics and clinical characteristics of the control cases (N = 20)**						
Gender		*M* 8			*F* 12	
Age	Total	*20–40 years*	*40–50 years*	*50–60 years*	*60–70 years*	> *70 years*
		2	3	4	6	5
No comorbidity known	2	1	1			
Arterial Hypertension	10			3	5	2
Malignancy	2			1		1
Chronic Kidney disease	3	1		1	1	
Overweight	3				2	1
Diabetes Mellitus	4		2			2
Chronic pulmonary disease	9			3	2	4

.

Tissue localization of SARS-CoV-2 was detected using antibodies against the nucleoprotein and the spike protein. Overall, we tested the hypothesis that the overexpression of proinflammatory cytokines plays an important role in the development of COVID-19-associated pneumonia by estimating the expression of multiple cytokines (IL-1β, IL-6, IL-10, IL-15, TNF-α, MCP-1), inflammatory cells (CD4, CD8, CD20, CD45), and fibrinogen.

### SARS-CoV-2 RNA detection

The viral titer in each specimen was estimated using real-time reverse transcription polymerase chain reaction (RT-PCR). Swabs of the upper respiratory tract (nasopharynx and oropharynx) were taken before the autopsy, whereas swabs of the lower respiratory tract (trachea and primary bronchi) were taken during the autopsy. Postmortem swabs were processed using the reagent system for SARS-CoV-2 RT-PCR (RealStar®, Altona Diagnostics, Germany). The limit of detection of the RT-PCR was 2000 copies of viral RNA/mL. RNA was quantitatively assessed to distinguish RNA of βCoV lineage B (B-βCoV) from that of SARS-CoV-2. For this, structural E-genes, specific for B-βCoV, and S-genes, specific for SARS-CoV-2, were amplified using RT-PCR, and the cycle threshold (Ct) values were used for analysis.

### Autopsies and tissue processing

Autopsies were performed in infection isolation rooms. Histological samples obtained after the autopsy were fixed in 10% buffered formalin for 48 h.

### Histological and immunohistochemical analysis

We performed routine hematoxylin–eosin staining for histopathologic evaluation of each sample. Immunohistochemical analysis to evaluate the distribution of SARS-CoV-2 in the tissue samples was performed on 5 µm thick paraffin-embedded sections of the brain (5 samples each case), heart (7 samples each case), lung (7 samples each case), trachea (1 sample each case), kidney (2 samples each case), liver (2 samples each case), spleen (1 samples each case), stomach (1 samples each case), gut (2 samples each case), thyroid (2 samples each case), and testicles (2 samples each case). We utilized anti-nucleocapsid (Santa Cruz Biotechnology, CA, USA) and anti-spike (Sino Biological, Germany) antibodies to detect viral particles. Lung sections were evaluated for the expression of multiple cytokines (IL-1β, IL-6, IL-10, IL-15, TNF-α, MCP-1), inflammatory cells (CD4, CD8, CD20, CD45), and fibrinogen. The dilution of antibodies and pretreatments for antigen retrieval are shown in Table 2.

**Table 2:** Antibodies, dilution and pretreatments for SARS-CoV-2 immunohistochemical study

Antibody	Producer	Dilution	Pretreatment	Incubation
IL-1β	Santa Cruz Biotechnology, Inc	1:200	HIER 0.1 M citrate buffer	2 h, 20 °C
IL-6	Santa Cruz Biotechnology, Inc	1:500	Proteinase K, 15 min. at 20 °C	2 h, 20 °C
IL-10	Santa Cruz Biotechnology, Inc	1:50	Proteinase K, 15 min. at 20 °C	2 h, 20 °C
IL-15	Santa Cruz Biotechnology, Inc	1:50	HIER 0.25 mM EDTA buffer	2 h, 20 °C
TNF-α	Santa Cruz Biotechnology, Inc	1:500	HIER 0.1 M citrate buffer	2 h, 20 °C
MCP-1	Santa Cruz Biotechnology, Inc	1:50	HIER 0.25 mM EDTA buffer	2 h, 20 °C
CD4	Santa Cruz Biotechnology, Inc	1:50	HIER 0.25 mM EDTA buffer	2 h, 20 °C
CD8	Santa Cruz Biotechnology, Inc	1:500	HIER 0.25 mM EDTA buffer	2 h, 20 °C
CD20	Santa Cruz Biotechnology, Inc	1:500	HIER 0.25 mM EDTA buffer	2 h, 20 °C
CD45	Santa Cruz Biotechnology, Inc	1:500	HIER 0.25 mM EDTA buffer	2 h, 20 °C
Fibrinogen	Santa Cruz Biotechnology, Inc	1:3000	Proteinase K, 15 min. at 20 °C	2 h, 20 °C
SARS-CoV-2 (nucleocapsid)	Santa Cruz Biotechnology, Inc	1:100	HIER 0.25 mM EDTA buffer	Overnight, 20 °C
SARS-CoV-2 (spike)	Sinobiological	1:1000	HIER 0.25 mM EDTA buffer	2 h, 20 °C

Primary antibodies were detected using a biotinylated secondary antibody and horseradish peroxidase-conjugated streptavidin (4plus HRP Universal Detection, Biocare Medical, CA, USA). 3,3’-Diaminobenzidine (DAB, Biocare Medical, CA, USA) and H2O2 (Betazoid DAB Chromogen Kit, Biocare Medical, CA, USA) were used as the chromogen and substrate, respectively. Subsequent counterstaining with hematoxylin–eosin allowed visualization of cell morphology and nuclei.

Cytokines, differentiation-related proteins, and fibrinogen were subjected to a semi-quantitative evaluation. Each slide was evaluated by 2 different investigators at × 40 magnification. The intensity of immunopositivity was assessed semi-quantitatively and expressed on a scale of 0–5 as follows: − , no immunoreactivity (0%); + /–, basal immunopositivity (5%); + , mild immunopositivity (10%); + + , isolated immunopositivity (33%); + + + , diffuse immunopositivity (66%), and + + + + , widespread immunopositivity (> 90%). In cases of divergent scores, a third investigator decided the final score.

### Ultrastructural examination

Lung, heart, and kidney samples were collected and assessed for the presence of viral particles using electron microscopy. Samples were fixed in glutaraldehyde, post-fixed in 1% osmium tetroxide, further processed according to standard transmission electron microscopy procedures, and embedded in Poly/Bed® 812 (Polysciences, Germany). Suitable thin sections were identified by toluidine blue staining and examined using a Zeiss EM-109 transmission electron microscope (Zeiss, Germany).

### Statistical analysis

Statistical analysis of the immunohistochemical experimental results, including those of the semi-quantitative estimation, were performed using the GraphPad Prism 8 software for Windows (GraphPad Software, CA, USA). The data was analyzed for normality using the Kruskal–Wallis test, followed by Dunn’s multiple comparisons test to compare the groups. For all statistical tests, a p-value < 0.05 was considered significant.

## Results

### RT-PCR analysis of swabs

Using RT-PCR, all swabs from Group 1 collected from the upper (nasopharynx and oropharynx) and/or the lower respiratory (trachea and primary bronchi) tracts before and during the autopsies, respectively, were positive for SARS-CoV-2. The control group was always negative on the swab result.

### Histopathological analysis

**Fig. 1:**
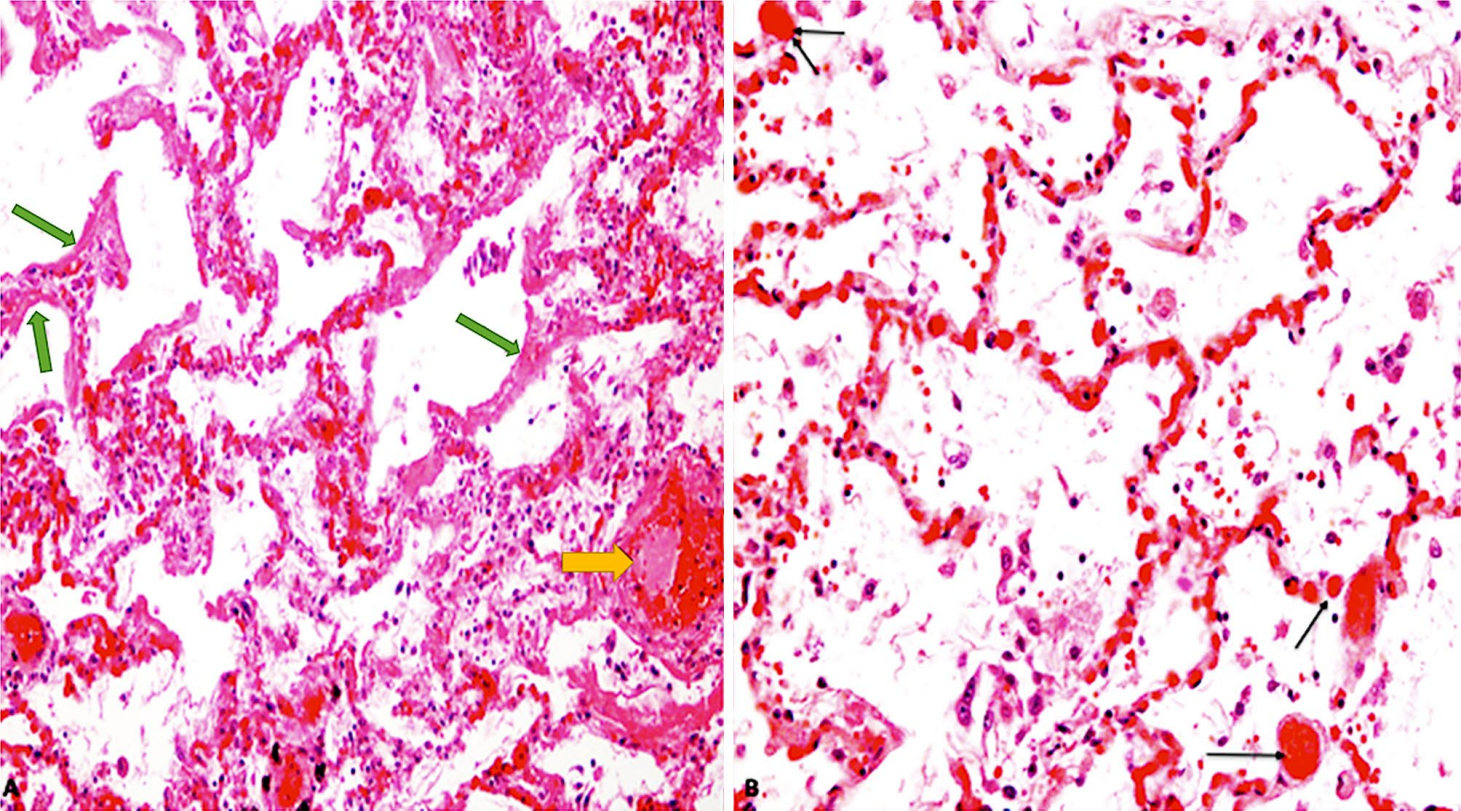
**A** Lung tissue showed edema, early stage DAD with hyaline membranes (green arrows) and microvascular thrombi (yellow arrows) (H&E, × 40); **B** Lung: capillary congestion, and microvascular thrombi (black arrows) (H&E, × 60)

Diffuse alveolar damage (DAD) was found in thirty-four cases; the main feature was exudative DAD, while in eighteen cases DAD was in the proliferative stage. We also observed desquamation of hyperplastic pneumocytes, presence of multinucleated cells, and foamy macrophages; there was also fibrosis and squamous metaplasia in advanced stages. Fourteen patients also had superimposed granulocyte focal confluent bronchopneumonia. The pulmonary vessel endothelia did not show vasculitis alterations, but the small arteries showed fibrin thrombi in twenty-three cases. (Fig. 1).

### Immunohistochemical analysis

Only some of the analyzed protein markers were significant for discriminating the COVID-19 group from the control group. IL-10 was excluded from further analyses since it did not exhibit any discriminatory power.

**Fig. 2:**
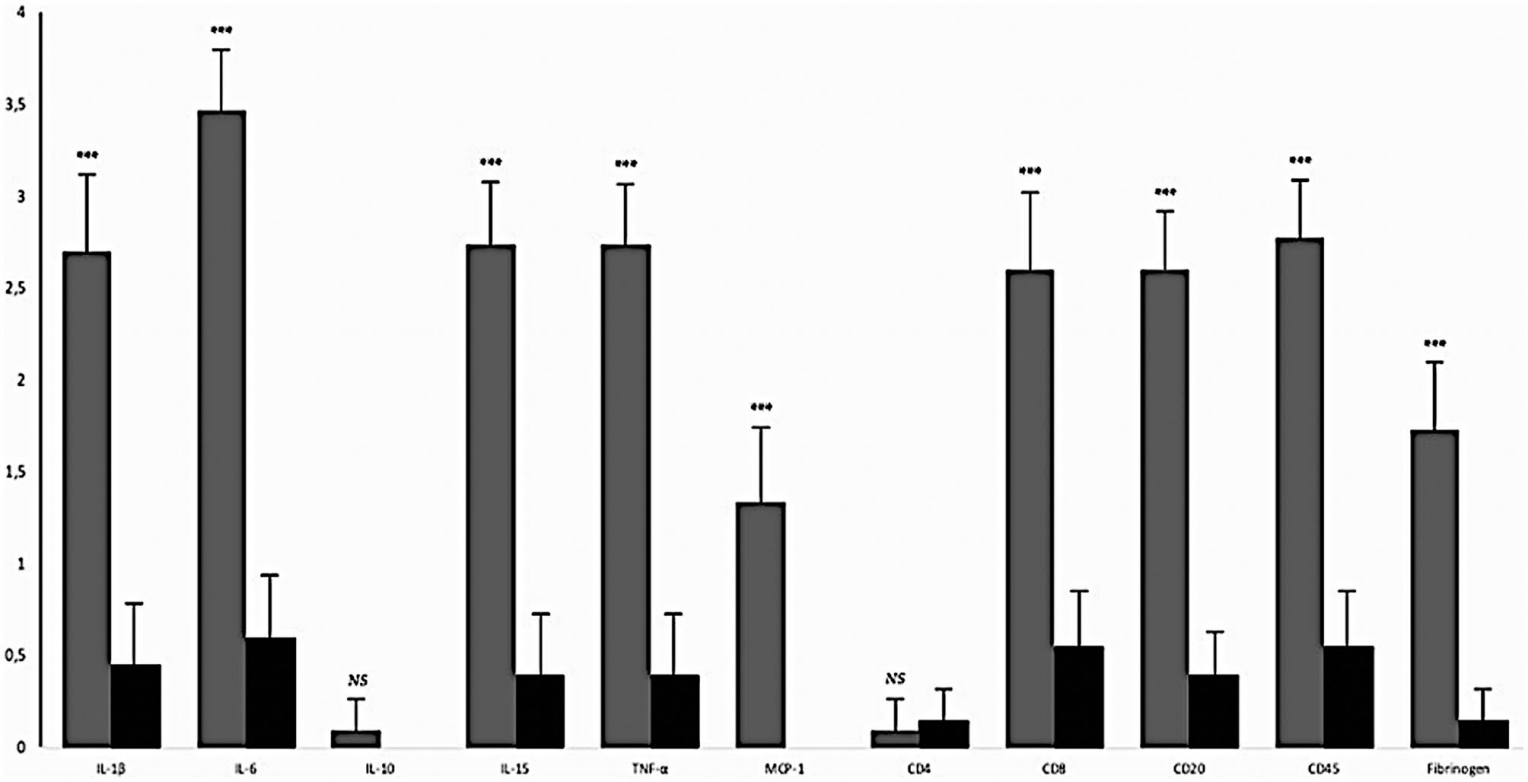
Statistically significant difference in the group of COVID-19-related death (grey columns) compared to the control group (black columns) for the following cytokines: IL-1β, IL-6, IL-15, TNF-α, MCP1, CD4, CD8, CD20, CD45, fibrinogen: *NS: p* > 0.05; **: p < 0.01; ***: p < *0.001*

Results of the immunohistochemical analyses, including the semi-quantitative analysis, are described using an ordinal scale, and the median value is reported. Morphometry-based microscopic analysis revealed that the immunohistochemical reaction generated by antibodies against TNF-α, IL-1β, IL-15, IL-6, MCP-1, CD8, CD20, and CD45 was significantly different between the COVID-19 group and the control group (Fig. 2). Results of the immunohistochemical analysis of lung specimens from the COVID-19 and control groups are presented in Tables 3 and 4.

**Table 3:** Expression and localization of SARS-CoV-2 antigen (nucleocapsid and spike) in principal organs using immunohistochemistry.^1^

	**Lungs and trachea**		**Liver**		**Heart**		**Kidney**		**Stomach and bowel**		**Spleen**	
	**Group 1**	**Group 2**	**Group 1**	**Group 2**	**Group 1**	**Group 2**	**Group 1**	**Group 2**	**Group 1**	**Group 2**	**Group 1**	**Group 2**
SARS-CoV-2 (nucleocapsid)	+ + +	-	+ + +	-	+ + + +	-	+ + + +	-	+ +	-	+ + +	-
SARS-CoV-2 (spike)	+ + +	-	+ + +	-	+ + + +	-	+ + + +	-	+ +	-	+ + +	-

^1^ The immunohistochemical findings and the gradation of the immunohistochemical reaction have been described with an ordinary scale and the median value has been reported. The reactions were graded as follows: 0 ( −) not expressed; 1 ( +) isolated and disseminated expression; 2 (+ +) expression in scattered foci; 3 (+ + +) expression in widespread foci; 4 (+ +  + +) widespread expression. The Group 1 are COVID-19-positive subjects and the Group 2 are controls

**Table 4:** Semi-quantitative evaluation and statistical analysis of the immunohistochemical findings and gradation of the immunohistochemical reaction in the lung samples.^1^

Antibody	Group 1 (COVID-19 positive)	Group 2 (controls)	Statistical value Group 1 vs. Group 2
IL-1β	+ + + +	+/-	***
IL-6	+ + + +	+/-	***
IL-10	+/-	-	NS
IL-15	+ + + +	+/-	***
TNF-α	+ + +	+/-	**
MCP-1	+ +	-	***
CD4	+/-	+/-	NS
CD8	+ +	+/-	***
CD20	+ +	+/-	***
CD45	+ +	+/-	***
Fibrinogen	+ +	-	***

^1^ Intensity of immunopositivity was assessed semi-quantitatively in the scale 0–5 as follows: − : no immunoreactivity (0%); ± : basal immunopositivity (5%); + : mild immunopositivity (10%); +  + : isolated immunopositivity (33%); +  +  + : diffuse immunopositivity (66%) and +  +  +  + : widespread immunopositivity (> 90%). NS: p > 0.05; **: p < 0.01; ***: p < 0.001

**Fig. 3:**
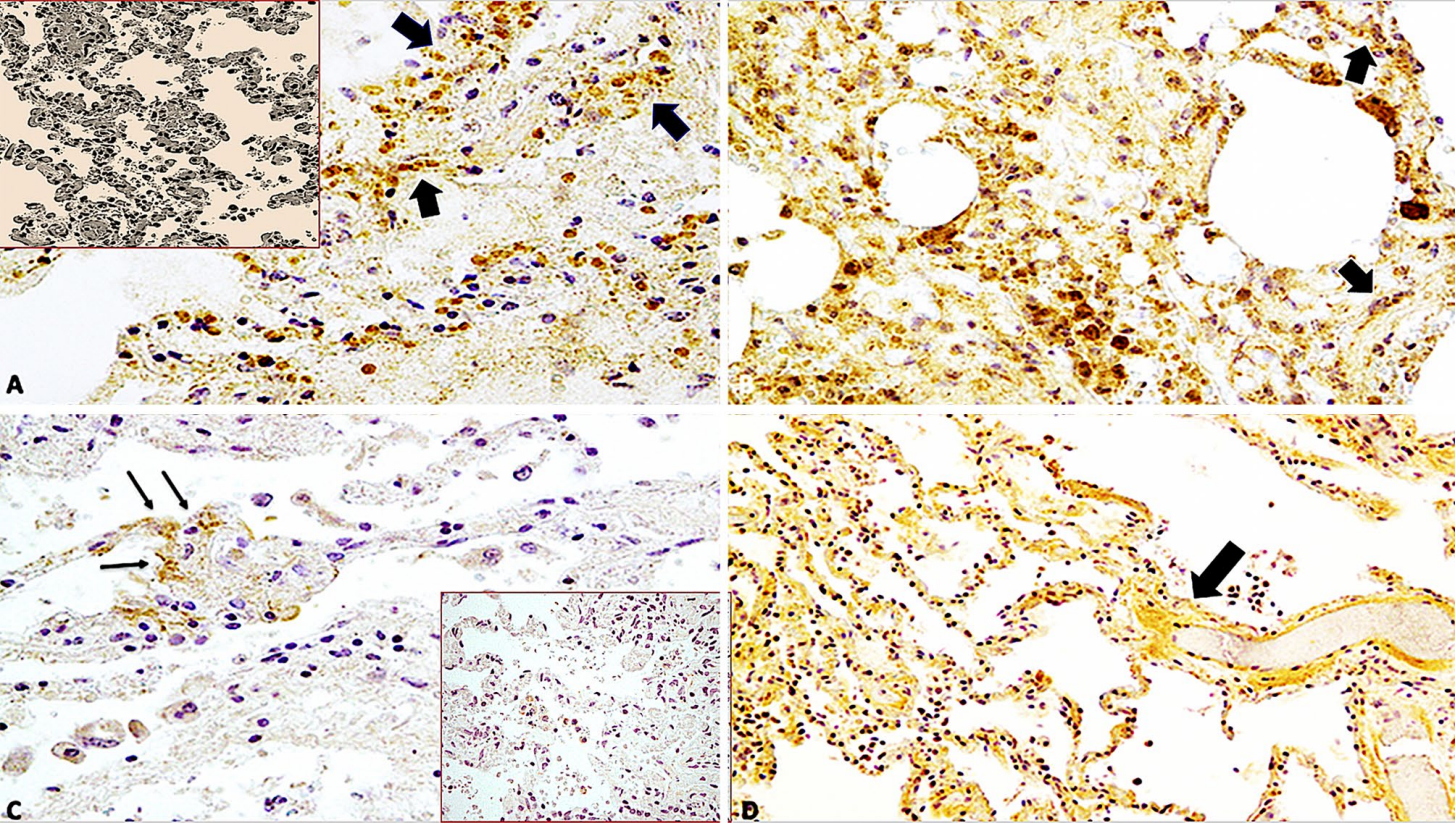
**A, B** Immunohistochemical reaction of IL-1β in group of COVID-19-related death showed a wide endothelial expression and positivity (brown reactions indicated with black arrows) in lung samples (× 60, × 100); Insert in (a): control case (× 60); **C, D** IL-6 showed a strong (black arrows) and diffusely positive reactions in capillary-alveolar endothelial cells in the COVID-19 group, expressed by brown dots in the endothelial cells than the negative control case (× 80, × 40); Insert in (c): control case (× 60)

**Fig. 4:**
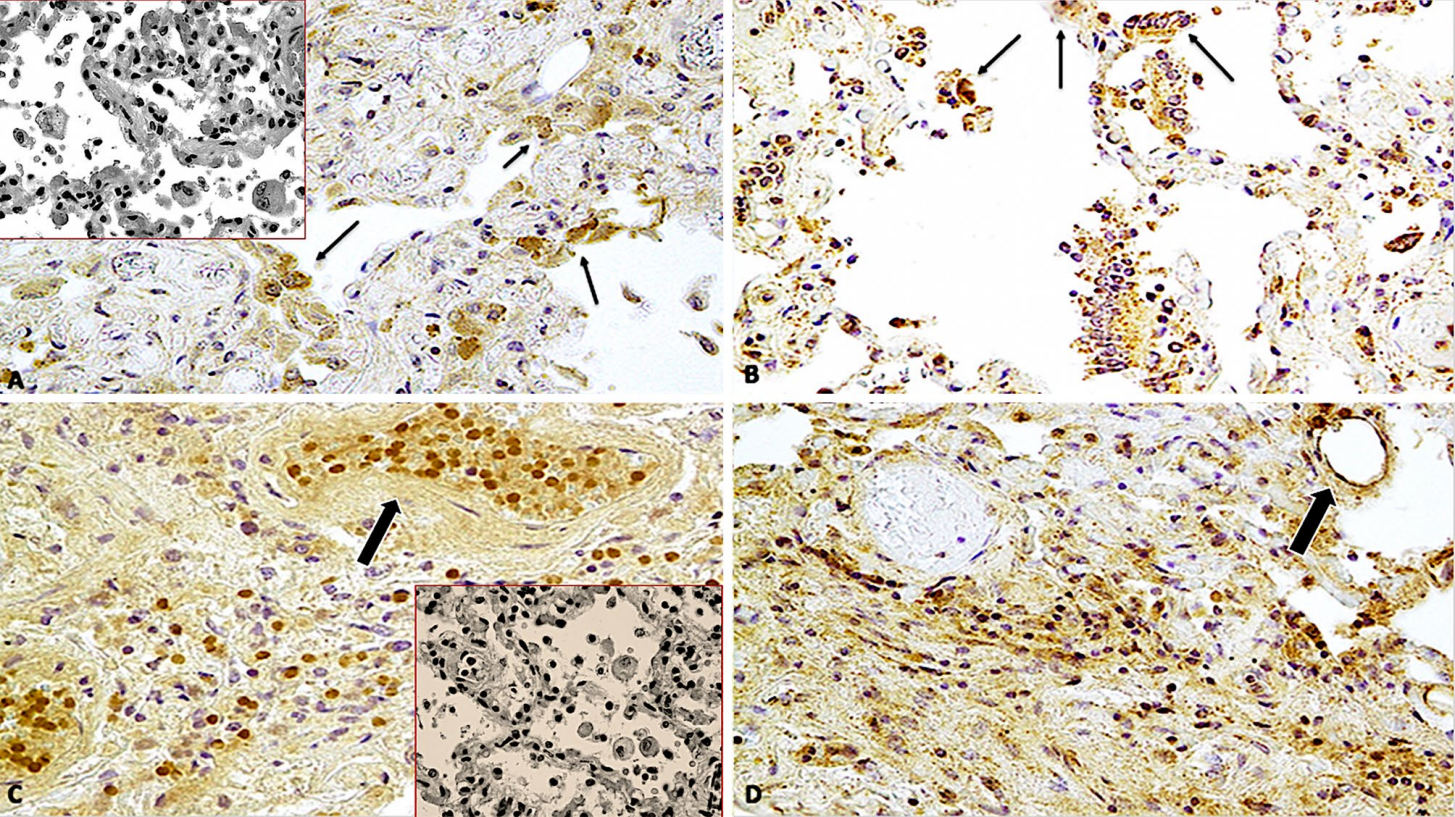
**A, B** Immunohistochemical reaction of IL-15 in group of COVID-19-related death showed a showed a strong positive reaction (arrows) in capillary-alveolar endothelial cells in lung samples (× 100, × 60); insert in (a): control case (× 60); **C, D** TNF-α showed a diffusely positive reaction in capillary-alveolar endothelial cells in the COVID-19 group, expressed by brown dots in the endothelial cells than the negative control case (× 100, × 60); insert in (c): control case (× 60)

**Fig. 5:**
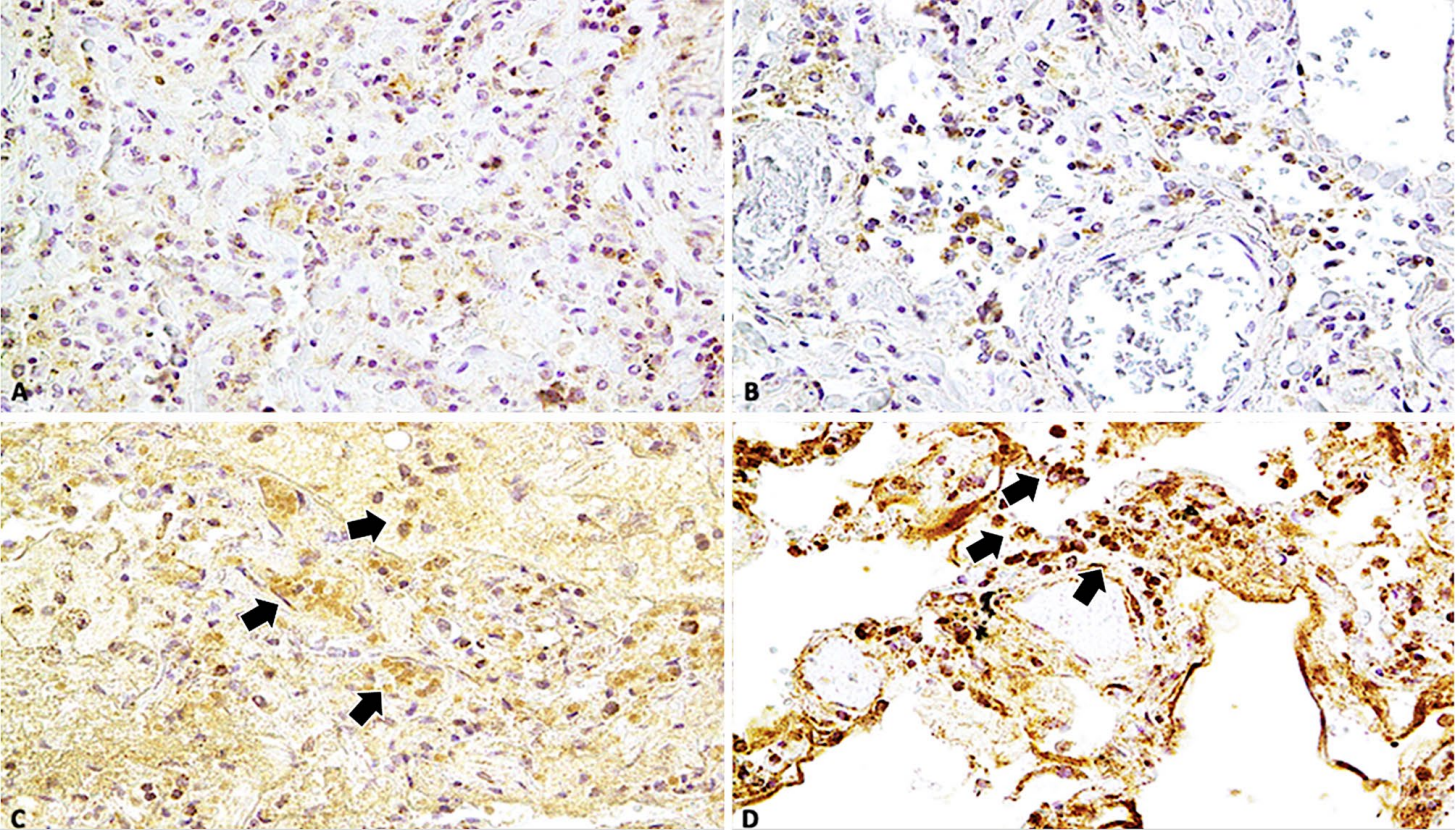
**A** Immunohistochemical reaction to CD4 + T cells demonstrated a greater positivity in lungs from control group (Group 2) than in COVID-19 group (Group 1); **B** (× 60, × 80); **C, D** CD8 + T cells were more numerous adjacent to the alveolar epithelial lining in the COVID-19 group than in the control group expressed by brown reactions in the endothelial cells than the negative control case (× 80, × 80)

Immunohistochemical staining showed that endothelial cells expressed IL-1β in lung samples obtained from patients of COVID-19 but not in those obtained from control individuals (p < 0.001) (Fig. 3A, B). Similarly, alveolar capillary endothelial cells showed strong and diffuse immunoreactivity for IL-6 (Fig. 3C, D) and IL-15 (Fig. 4A, B), observed as red dots in the cytoplasm, in the COVID-19 group but not in the control group (p < 0.001). In all twenty-three cases, the finding of microthrombi in the pulmonary vessels correlated with intense positivity to the immunohistochemical reaction with pro-inflammatory cytokines (IL-1 β, IL-6, TNF- α). TNF-α showed a higher immunoreactivity in the COVID-19 group (Fig. 4C, D) than in the control group (p < 0.01). CD8 + T cells were more numerous in the lung samples obtained from COVID-19 patients than in those obtained from control individuals (p < 0.01), whereas the number of CD4 + T cells present adjacent to the alveolar epithelial lining was lesser in the lung samples obtained from COVID-19 patients than in those obtained from control individuals (Fig. 5).

**Fig. 6:**
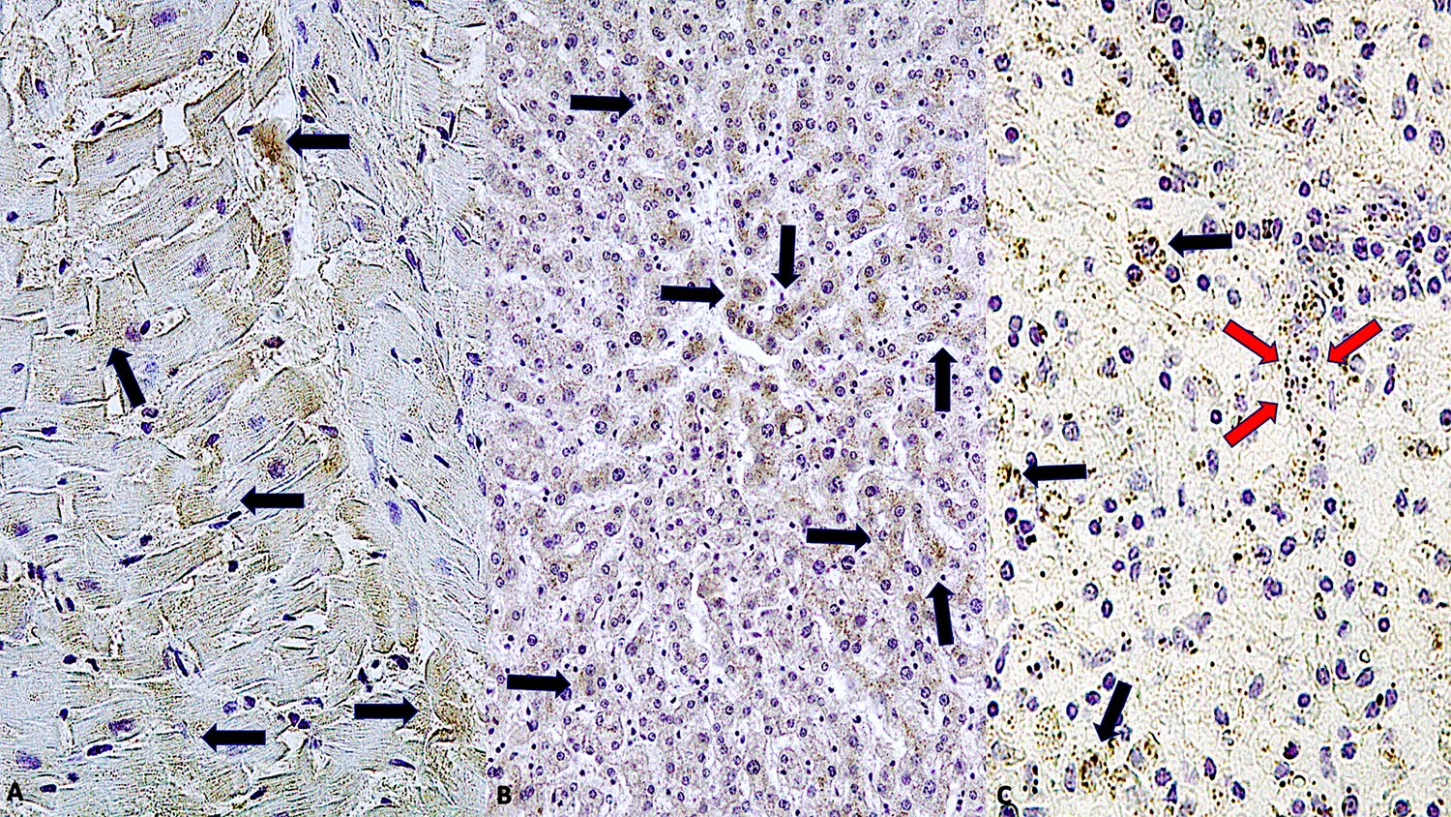
**A** Heart: immunohistochemistry demonstrated strong reactions (arrows) into the myocardiocytes (× 100); **B, C** Liver cells colonized by numerous viral particles (arrows); diffuse positivity (arrows) to SARS-CoV-2 antigen [nucleocapsid (black arrows) and spike (red arrows)] into the spleen (× 100)

Expression and localization of SARS-CoV-2 antigen (nucleocapsid and spike) were demonstrated in principal organs such as heart, liver and spleen using immunohistochemistry (Fig. 6).

### Ultrastructural results

**Fig. 7:**
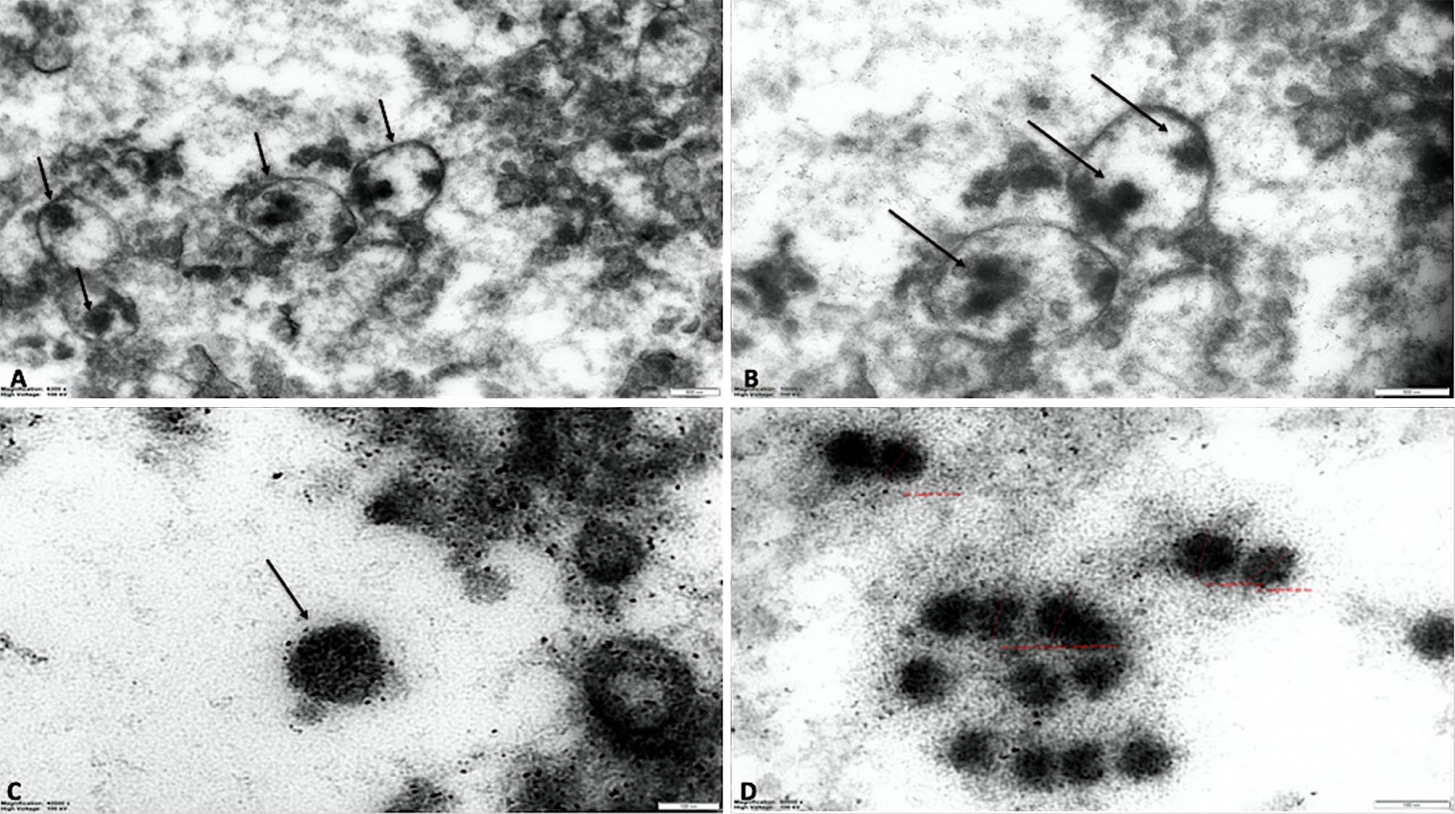
**A, B** Lung: transmission electron microscopy demonstrated viral particles into the endothelial cells (× 6300, × 10,000; bar 500 nm); **C, D** Glomerular endothelial cells colonized by numerous viral particles (× 40,000, × 50,000; bar 100 nm)

Electron microscopy revealed viral particles within the cells of the lungs, trachea, kidney, and large intestine (Fig. 7A, B) in the COVID-19 group. Renal glomerular endothelial cells exhibited free viral particles (74–82 nm in diameter) localized in the cytoplasm or within a vacuole (Fig. 7C, D).

## Discussion

Current evidence suggests that a "cytokine storm" is the major cause of ARDS and multiple organ failure, and it has been consistently linked with fatal outcomes [81, 82]. Activated white blood cells, B cells, T cells, natural killer (NK) cells, macrophages, dendritic cells, neutrophils, monocytes, and resident tissue cells, such as epithelial and endothelial cells, release large amounts of proinflammatory cytokines. High levels of proinflammatory cytokines, such as IL-1, IL-6, IL-7, IL-12, IFN-γ, TNF-α, IP-10, MIP-1A, MCP-1, GCSF, and IP-10, have been observed in COVID-19 patients and are generally associated with severe lung damage [83–85]. Activated resident macrophages and pneumocytes initiate an inflammatory response triggered by the presence of SARS-CoV-2 in the lungs, leading to the overproduction of proinflammatory cytokines and chemokines, which are involved in endothelial cell apoptosis, increased vascular permeability, pulmonary exudation, hypoxia, and multiple organ failure [86]. Overproduction of cytokines is related to the development of clinical symptoms. For example, IFN-γ can cause fever, chills, headaches, dizziness, and fatigue; TNF-α is associated with flu-like symptoms [87]; and IL-6 is associated with activation of the complement and coagulation cascade, which leads to diffuse intravascular coagulation (DIC). IL-6 also promotes myocardial dysfunction [88]. Together with reactive oxygen species, IL-6, IL-8, IL-1β, GM-CSF, and other chemokines cause ARDS, leading to pulmonary fibrosis and death. In the early stages of the infection, a hyper-inflammatory state is followed by an immunosuppressed state, and this is potentially associated with a decrease in CD4 + and CD8 + T cells [89]. COVID-19 patients are characterized by a distinct decrease in memory T cells and cytotoxic CD8 + T cells. A decrease in total lymphocytes (CD4 + and CD8 + T cells, B cells, and NK cells) has also been reported [80]; however, the mechanism of lymphopenia is unclear and needs to be investigated further. It has been hypothesized that a direct infection of T cells with SARS-CoV-2 triggers a cytopathic effect; however, the lack of ACE-2 receptors on the lymphocytes seems to exclude the possibility of a direct injury and indicates that SARS-CoV-2 infects human T cell lines through the CD147 spike protein on the surface of T lymphocytes, leading to cell apoptosis [90]. The dysfunction of lymphocytes impairs the adaptive immune response of the host, and an uncontrolled viral infection leads to the increased macrophage infiltration, further worsening the damage to the lungs. Finally, the spread of the virus in the bloodstream directly impacts other organs and leads to a dysfunction of the systemic microcirculation, while the systemic inflammatory response causes viral sepsis. Some authors have proposed the role of neutrophils in the exacerbation of the host response to SARS-CoV-2, wherein they trigger a cascade of inflammatory reactions that facilitate micro-thrombosis and result in damage to the pulmonary, cardiovascular, and renal systems [70, 80, 91].

Clinical characteristics of patients infected with SARS-CoV-2, such as pneumonia, ARDS, sepsis, and multiple organ failure, provide evidence for the fact that the ACE-2 receptors on the ciliated cells of the airway epithelium and alveolar type II cells are the route of viral entry. It is well known that the coronavirus spike protein has 2 domains, S1 and S2. The S1 domain binds to the host ACE-2 receptor, while the S2 domain is responsible for cell membrane fusion. The inflammatory response induced by a viral infection is critical to inhibiting viral replication; however, an excessive immune response could be crucial to the pathogenesis of a disease. The interaction between the spike protein and ACE-2 receptor leads to the downregulation of ACE-2, resulting in the local enhancement of angiotensin II production and unrestricted stimulation of the angiotensin receptor (AT1-R). Additionally, binding of ACE-2 receptor with the SARS-CoV-2 spike glycoprotein induces the formation of syncytial multinucleated cells.

Studies involving cadavers are often limited to a single case or minimally invasive approaches, such as biopsies and thoracotomies, and those involving a large number of cases remain a rarity (Table 2). DAD (exudative/proliferative) with interstitial lymphocytic infiltration and atypical large pneumocytes has been reported in some cases of COVID-19. Mild infiltration of interstitial mononuclear inflammatory cells has been occasionally observed in cardiac samples, and the neuroinflammatory response to COVID-19 is still debated. Spleen atrophy, lymph node necrosis, focal hemorrhage, and infiltration of inflammatory cells in the kidney and liver have been reported, demonstrating the impact of the SARS-CoV-2 infection on multiple organs [42, 54, 56, 63, 72, 73]. Immunohistochemistry is crucial in postmortem investigations, and immunohistochemical staining for various inflammatory cells, such as lymphocytes, macrophages, neutrophils, and endothelial cells, is generally performed in autopsy studies [43, 50–52, 60, 65, 77]. On the other hand, electron microscopy allows for the visualization of intracellular viral particles with distinctive spikes and solar corona distribution [44, 46, 62, 66–70, 75, 92].

### Lungs and the respiratory system

Clinical studies have reported a correlation between elevated serum levels of proinflammatory cytokines, such as IFN-γ, TNF-α, IL-6, and IL-8, and lung injury and poor prognosis [93]. Postmortem specimens of SARS-CoV-2-infected lungs exhibited histological features of DAD with necrosis of the alveolar lining, hyperplasia of type II pneumocytes, intra-alveolar fibrin deposition, mild interstitial edema, and infiltration of lymphocytes in the perivascular space in the early phase of the infection [43, 44, 51, 65, 70, 72]. Our results confirm the presence of these histological findings in all cases examined. A recent report has described the following 3 fundamental characteristics that connote pulmonary changes induced by COVID-19: severe endothelial injury associated with the intracellular localization of SARS-CoV-2 and disruption of endothelial cell membrane, widespread vascular thrombosis with microangiopathy and occlusion of alveolar capillaries, and growth of new vessels via intussusceptive angiogenesis [50]. Another report also suggested that the magnitude of cytokine secretion is associated with severity of COVID-19 and that postmortem lung samples showed higher endothelial expression of IL-6 and TNF-α in the diseased condition than in control condition. These pathological modifications reflect endothelial dysfunction, which when combined with pyroptosis can lead to systemic thrombosis [94].

Our findings support this. Immunohistochemical evaluation of pro-inflammatory cytokines showed a high and widespread lung expression of IL1β, IL-6, IL-15 and TNF-α in deaths caused by severe COVID-19 pneumonia. We also found an increase in MCP-1 expression compared to controls, which is consistent with local macrophage activation.

Infiltration of inflammatory cells, represented by CD4 + and CD8 + lymphocytes in the perivascular space, ranged from scarce to moderate according to disease severity. Some authors have described a paucity of CD8 + , CD57 + , and NK cells in the alveolar septa, lymphoid organs (spleen and lymph nodes), and peripheral blood of COVID-19 patients [43, 70]. A paucity of CD20 + B cells has also been reported in a few cases.

Our results do not confirm these data. The immunohistochemical investigations carried out in our study revealed the presence of abundant lymphocytes (CD45 +) in the alveolar septa. These were mainly identified as CD8 + T lymphocytes and B (CD20 +) lymphocytes. CD4 + T lymphocytes were few in number, even less numerous than in the control group (difference not statistically significant). This difference could be attributed to the clinical history of our cases: almost all subjects died outside a hospital setting and therefore did not receive specific or symptomatic therapies that could have modulated the inflammatory response [95].

CD68 + macrophages and atypical giant cells have been observed in alveolar spaces in COVID-19 patients, and type II pneumocytes with the enlarged, bright, eosinophilic nucleoli have been reported to increase in size in the diseased condition [70]. Intraluminal CD61 + megakaryocytes, associated with the production of fibrin and platelets, exhibited nuclear hyperchromasia and atypia in COVID-19 patients [60]. In some cases of COVID-19, superimposed bronchopneumonia has been reported, and vascular thrombosis with microangiopathy and occlusion of alveolar capillaries has also been observed in lung samples of patients with COVID-19 [63]. Immunohistochemical investigations showed a higher number of ACE-2-positive cells in the lungs of patients with COVID-19 than that in uninfected controls. Studies have reported that Ki-7 was expressed in alveolar and bronchiolar cells, indicating a high index of epithelial cell proliferation, in COVID-19 patients [43]. Squamous metaplasia in the distal airways and alveoli refers to the proliferation of bronchiolar basal cells in response to an epithelial injury. Electron microscopic analysis has revealed significant changes in endothelial morphology, including disruption of intercellular junctions, cell swelling, and a loss of contact with the basal membrane, in COVID-19 patients. Round viral particles were also observed in the tracheal, bronchial, type I, and type II alveolar epithelial cells in COVID-19 patients.

### Heart and cardiovascular system

The mechanisms underlying the pathogenesis of SARS-CoV-2 in patients with cardiovascular comorbidities are still not completely understood. Increasing cardiac stress due to respiratory failure and hypoxemia, myocardial viral infection, indirect injury from a systemic inflammatory response, or a combination of all 3 mechanisms has been proposed as the underlying mechanism [96–98]. It has been hypothesized that the downregulation of ACE-2 in response to a SARS-CoV-2 infection leads to myocardial dysfunction, potentiating angiotensin II release and AT1-R stimulation [99].

In the literature, papers highlight the presence of vascular endotheliitis, thrombosis and angiogenesis in COVID-19. Endothelial and platelet dysfunction are considered important players in the multifactorial pathogenesis of COVID-19-associated coagulopathy [61].

The mechanisms involved can be summarised as follows:

1. Endothelial cell dysfunction and apoptosis could lead to basal membrane exposure resulting in macro- and/or micro-thrombotic angiopathy.
2. Endothelial cell damage due to ATII upregulation would underlie organ or generalised vasoconstrictive responses.
3. Endothelial cells have often been recognised as part of the innate immune system and could be responsible for an exacerbated response in the inflammatory phase of the disease.
4. Microangiopathy could be supported locally by an endotheliitis demonstrated by perivascular inflammatory infiltrates.
5. Generalised cellular hypoxia may finally result from mitochondrial dysfunction conducting to endothelial cell dysfunction [100].

The damaging action may occur through tissue factor (TF) activation, which occurs mainly in the endothelium, platelets and perivascular cells. Due to the increase in angiotensin 2 (AT2) in response to down-regulation of angiotensin-converting enzyme 2 (ACE-2), TF overexpression is most likely a trigger for TF activation [101]. The SARS-CoV-2 virus uses ACE2 and transmembrane serine protease 2 (TMPRSS2) to infect cells. The balance between circulating ACE2 and membrane-bound ACE2 receptor is therefore crucial to prevent SARS-CoV-2 entry into target cells, a mechanism dependent on membrane-bound ACE2 receptors [102].

It is interesting to stress the importance of pericytes. These perivascular cells are responsible for maintaining the integrity of microvessels and show high expression of the ACE2 receptor. The authors hypothesised that the decrease in pericytes and the apoptotic mechanism due to direct damage by SARS-COV-2 could be the initial trigger of microvasculopathy [103]. The virus affects not only the epithelial cells of the lung parenchyma via ACE2, but also endothelial cells throughout the body, thus leading to generalised endothelial damage and inflammation, so-called endotheliitis. In patients with severe COVID-19 infection, endotheliitis is demonstrated both by an increase in the number of circulating endothelial cells and by elevated levels of soluble endothelial cell adhesion molecules and inflammatory cytokines [104]. In post-mortem studies, the accumulation of lymphocytes, plasma cells and macrophages under the endothelial cells and in the perivascular spaces is described [61]. Histological findings from both in vivo biopsies and post-mortem investigations, showed lymphocytic endotheliitis with apoptotic bodies and viral inclusion in various organs. Severe inflammation with endotheliitis may lead to disseminated intravascular coagulation with subsequent thrombosis of small or large vessels [98]. Pathological findings such as cell swelling, severe endothelial injury, disruption of intercellular junctions and loss of basement membrane contact in COVID-19 patients indicate the progression from activation to dysfunction until destruction of endothelial cells, which, supported by vascular endotheliitis, leads to the formation of capillary microthrombi [50, 105].

Myocardial damage and heart failure have been reported to be associated with high levels of troponin I and B-type natriuretic protein and high mortality rates [106]. There are no studies that have demonstrated the presence of SARS-CoV-2 within the myocardial tissue, and reports of infiltration of the myocardium by macrophages and CD4 + T lymphocytes is limited to single case studies [25, 62, 107].

In the evaluation about the localization of the virus, we detected the presence of cytoplasmic inclusions in the heart stained with both anti-SARS-CoV-2 antibodies used by us. We interpreted the result as the presence of the virus in myocardiocytes. It is emphasized that myocarditis or signs of local inflammation were not detected in any case in our study.

Viral particles have been observed within endothelial cells using electron microscopy, demonstrating the direct cellular impact of SARS-CoV-2 on the cardiovascular system [108]. Direct endothelial cell damage is associated with the dysregulation of vascular tone and homeostasis, microvascular vasoconstriction and ischemia, and a pro-coagulant state. Ischemic injury of myocytes has been reported by Buja et al. [68] and Menter [44]. Arrhythmias, such as tachycardia, bradycardia, and asystole, have also been reported in COVID-19 patients and are associated with hypoxemia, metabolic derangements, systemic inflammation, or myocarditis. A possible role of the prothrombotic state has been hypothesized in COVID-19 patients exhibiting acute coronary syndromes and myocardial infarction [109].

It has been hypothesized that a thrombophilic state could be induced as a result of SARS-CoV-2 infection via the activation of the coagulation system. Prothrombin time and activated partial thromboplastin time are increased during activation of coagulation and decreased in cases of consumptive coagulopathy in patients with COVID-19; fibrinogen expression is also increased in these patients [110]. Further, the thrombin-antithrombin complex, fibrin-degradation products and D-dimers were found to be increased in the late stages of the disease. In addition, platelet counts decreased in the late stages of the infection. The mechanism under DIC in COVID-19 patients has not been clearly identified, and infection-induced coagulopathy and secondary hyperfibrinolysis are hypothesized to be involved [111]. In a study by Wichmann et al. [63], of the 12 autopsies performed, deep vein thrombosis occurred in 58% of the cases, and in one-third of these, pulmonary thromboembolism was the cause of death. Fatal pulmonary thromboembolism has also been described by Lax et al. [56]. A causal relationship between the inflammatory and reparative process, involving DAD, is hypothesized to lead to endothelial damage [62, 112].

### Renal system

The impact of a SARS-CoV-2 infection on the renal system has been reported in a clinical study, wherein the patients exhibited proteinuria, hematuria, elevated blood urea nitrogen levels, and acute kidney injury [69]. In a study by Pei et al. [113], a higher mortality was observed in the early stages of infection in patients who exhibited symptoms of renal involvement. The severity of pneumonia triggered by the SARS-CoV-2 infection is a risk factor for acute kidney injury in COVID-19 patients. Ischemic injury, cytokine storm, and direct viral infection are plausible mechanisms of renal injury in COVID-19. In addition, acute tubular necrosis, loss of brush border cells, vacuolar degeneration, dilatation of the tubular lumen with cellular debris and necrosis, and detachment of the epithelium from the tubular basement membrane have been observed via light microscopy in patients with COVID-19 [44]. Immunohistochemical staining revealed an altered ACE-2 expression pattern in the kidneys of COVID-19 patients, with elevated expression in proximal tubular cells in areas of severe acute tubular injury. The tubular and glomerular visceral epithelial cells of the kidney are the main targets of SARS-CoV-2, while the endothelium seems to be excluded from direct damage. In the kidneys, ACE-2 is expressed in the apical brush border cells of the proximal tubules and in the podocytes, while it is not expressed in the endothelial cells. This peculiar distribution may explain the presence of viral particles in the tubular epithelium and podocytes in autopsy samples subjected to electron microscopic analysis [69, 92]. Recently, the involvement of CD147, a transmembrane glycoprotein that is highly expressed on the surface of proximal tubular epithelial cells and infiltrating inflammatory cells and is targeted by SARS-CoV-2, in diseases of the kidney has been hypothesized. Ischemic changes with accumulation of plasma in Bowman’s space have also been reported in some COVID-19 cases.

## Conclusions

Our study highlights the morphological impact of the cytokine storm triggered by SARS-CoV-2 infection and the potent inflammatory response involved in the pathogenesis of COVID-19. The cytokines involved are a complex group of mediators, particularly proinflammatory cytokines such as IL-1β, IL-6, IL-15, and TNF-α, which are produced at sites of tissue inflammation [83, 114, 115].

We have experimentally confirmed that there is a specific immune response, with a cytokine storm linked to coagulopathy [53]. Further autopsy studies are needed to expand this evidence and highlight the pathognomonic signs of the disease, as well as to facilitate the establishment of standard practices for collection of autopsy and postmortem data [116, 117].

## Key points

1. The aim of this study was to clarify the correlation between infection due to SARS-COV-2 and the inflammatory response, and to investigate the expression of cytokines such as TNF-α, IL-1β, IL-6, MCP-1, IL-10, IL-15, and leukocyte markers (CD 4, CD 8, CD20, CD 45) in cases of COVID-19 deaths.
2. Our study highlights the morphological impact of the cytokine storm triggered by SARS-CoV-2 infection and the potent inflammatory response involved in the pathogenesis of COVID-19.
3. The cytokines involved are a complex group of mediators, particularly proinflammatory cytokines such as IL-1β, IL-6, IL-15, and TNF-α, which are produced at sites of tissue inflammation.
4. Post the cytokine storm, the virus targets organs that express ACE-2, such as the lungs, heart, and kidneys.
